# The Role of Non-Human Sialic Acid Neu5Gc-Containing Glycoconjugates in Human Tumors: A Review of Clinical and Experimental Evidence

**DOI:** 10.3390/biom15020253

**Published:** 2025-02-10

**Authors:** Rancés Blanco, Juan P. Muñoz

**Affiliations:** 1 Investigador Independiente, Av. Vicuña Mackenna Poniente 6315, La Florida 8240000, Chile; rancesblanco1976@gmail.com; 2 Laboratorio de Bioquímica, Departamento de Química, Facultad de Ciencias, Universidad de Tarapacá, Arica 1000007, Chile

**Keywords:** N-glycolylneuraminic acid, Neu5Gc, Neu5Gc-GM3 ganglioside, sialic acid, cancer

## Abstract

N-Glycolylneuraminic acid (Neu5Gc) is a sialic acid variant commonly found in most mammals but not synthesized by humans due to an inactivating mutation in the CMP-Neu5Ac hydroxylase (*CMAH*) gene. Despite this, Neu5Gc-containing molecules are consistently detected in human tissues, particularly in malignant tumors. However, the mechanisms underlying Neu5Gc accumulation and its role in cancer development remain poorly understood. Objectives: This review aims to analyze clinical and experimental evidence regarding the presence of Neu5Gc-containing glycoconjugates in both tumor and non-tumor human tissues, exploring potential mechanisms of the Neu5Gc expression and evaluating its contribution to tumor biology, with a particular focus on the Neu5Gc-GM3 ganglioside. Methods: A comprehensive review of the literature was conducted, integrating findings from immunohistochemistry, chromatography, and molecular studies to assess the expression and implications of Neu5Gc in cancer biology. Results: Neu5Gc-containing glycoconjugates were found to preferentially accumulate in various malignant tumors, while their presence in normal tissues was restricted to cells with high turnover rates. This accumulation is potentially mediated by dietary uptake, hypoxic conditions, and metabolic alterations in cancer cells. Additionally, Neu5Gc-containing molecules were associated with the activation of oncogenic pathways. Conclusion: Neu5Gc-containing glycoconjugates play a multifaceted role in cancer progression and present potential as prognostic markers and therapeutic targets.

## 1. Introduction

**Figure 1:**
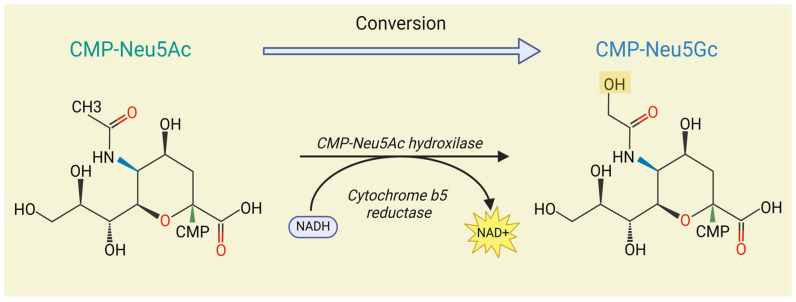
Enzymatic conversion of CMP-N-acetylneuraminic acid (Neu5Ac) to CMP-N-glycolylneuraminic acid (Neu5Gc) residue by CMP-Neu5Ac hydroxylase (CMAH). Created with Biorender.com (accessed on 24 November 2024).

Sialic acids are a family of acidic nine-carbon sugars typically located at the terminal residues of glycan chains on the vertebrate cellular glycocalyx and on secreted glycoproteins [1,2]. Due to their prominent position on cell surfaces, sialic acids play a pivotal role in various pathological processes in cancer, including cell signaling, tumor dissociation, invasion, cell–matrix interactions, angiogenesis, immune modulation, and metastasis formation [3]. In mammals, there are two predominant forms of sialic acids: N-acetylneuraminic acid (Neu5Ac) and N-glycolylneuraminic acid (Neu5Gc). The structural difference between these two variants is a single oxygen atom at the C-5 position in Neu5Gc, a modification catalyzed by cytidine monophosphate-N-acetylneuraminic acid hydroxylase (CMP-Neu5Ac hydroxylase, CMAH) [4] (Figure 1). Despite being a subtle difference, this single oxygen atom significantly influences many aspects of cellular behavior [5,6].

The synthesis of sialylated glycoconjugates comprises about 20 sialyltransferases grouped in the ST3Gal, ST6Gal, ST6GalNAc, and ST8Sia families, which catalyze the transfer of sialic acid from a donor substrate (CMP-sialic acid) to a glycan acceptor.

While neuraminidases (NEU1–4) are responsible for the cleavage of sialic acid residues from glycan chains, regulating cell-surface sialylation [7], the released sialic acids that resulted from neuraminidase activity can be reused by cells in the synthesis of new CMP-sialic acids [8]. In cancer, the upregulation of sialyltransferases, the downregulation of neuraminidases, and/or the increased availability of CMP-sialic acid lead to the hypersialylation on the cell surface [7,9]. The hypersialylation in cancer cells involved a variety of oncogenes, including *RAS* and *MYC*, and it is also related to tumor hypoxia [10,11,12]. For instance, the gangliosides, a subclass of glycosphingolipids, exemplify the complex sialylated structures involved in cancer. These molecules are synthesized from lactosylceramide (LacCer) or galactosylceramide (GalCer) precursors through sequential enzymatic actions catalyzed by various sialyltransferases (Figure S1).

On the other hand, human cells predominantly express Neu5Ac, as they have lost the ability to synthesize Neu5Gc due to an exon deletion and frameshift mutation in the *CMAH* gene [13]. Despite this genetic limitation, the aberrant expression of Neu5Gc-containing molecules (such as glycoproteins and glycosphingolipids) is frequently observed in various human tumors, while normal tissues generally exhibit only trace levels of these glycoconjugates [14,15,16]. Malignant cells are known to acquire Neu5Gc through exogenous uptake, incorporating it into newly synthesized glycoconjugates. This uptake is potentially augmented by the hypoxic conditions characteristic of the tumor microenvironment [17,18,19]. In addition, alternative pathways for Neu5Gc synthesis from other metabolic intermediates in some malignant cells were suggested [18,20].

Experimental and clinical studies indicate that Neu5Gc-containing glycoconjugates contribute significantly to tumor development and progression [21,22,23,24]. One of the proposed mechanisms involves the chronic inflammatory response triggered by the co-occurrence of Neu5Gc and circulating anti-Neu5Gc antibodies, which may promote tumorigenesis [25,26]. Consequently, Neu5Gc-containing molecules are regarded as tumor-associated antigens and have become promising targets for cancer immunotherapy. For instance, encouraging results have been reported in breast cancer, cutaneous melanoma, and non-small cell lung cancer (NSCLC) patients treated with molecular vaccines targeting the Neu5Gc-containing GM3 ganglioside (Neu5Gc-GM3) [27,28,29]. However, the biological implications of the Neu5Gc expression in human tissues, particularly in the context of cancer, remain incompletely understood.

This review aims to provide a comprehensive analysis of the current clinical evidence regarding the presence of Neu5Gc in both normal and malignant human tissues. We also explore the mechanisms underlying Neu5Gc expression in cancer and discuss its contribution to tumor development and progression, with a particular focus on Neu5Gc-GM3.

## 2. Occurrence of Neu5Gc-Containing Glycoconjugates in Human Tissues

The distribution and expression of Neu5Gc-containing glycoconjugates vary significantly between different human tissues, influenced by factors such as developmental stage, tissue type, and cellular turnover rates. The following sections delve deeper into the occurrence of Neu5Gc in human tissues, focusing on its expression in fetal and normal adult tissues, as well as its aberrant accumulation in malignant tumors. Understanding these patterns is essential for elucidating the biological significance of Neu5Gc in health and disease.

### 2.1. Expression of Neu5Gc in Fetal and Normal Adult Tissues

The presence of Neu5Gc-containing glycoconjugates has been demonstrated in both fetal and normal adult tissues, with significant differences in their distribution and expression patterns. Immunohistochemistry (IHC) using anti-Neu5Gc antibodies has revealed Neu5Gc expression in gastric epithelial cells and their secretions, vascular endothelium, and the placenta [30]. Specifically, Neu5Gc was detected in placental blood vessel endothelial cells [31].

In fetal tissues, Neu5Gc-containing gangliosides, including Neu5Gc-GM3, were identified in various organs. Two-dimensional thin-layer chromatography followed by immunostaining showed Neu5Gc-GM3 in one-third (33.3%) of fetal intestine samples and in meconium [32]. Additionally, IHC with the P3 monoclonal antibody (mAb), which reacts with Neu5Gc-GM3, other Neu5Gc-gangliosides, and sulfated glycolipids, highlighted its expression across multiple fetal organs, including the esophagus (80.0%), stomach (50.0%), small intestine (100%), large intestine (50.0%), trachea (80.0%), lung (50.0%), suprarenal gland (80.0%), and cerebellum astrocytes (50.0%) [33]. Further studies using the 14F7 mAb, which is highly specific for Neu5Gc-GM3, confirmed its presence in fetal tissues from 12- to 18-week-old fetuses. Expression was noted in the stomach, small and large intestine, liver, and kidney (4/4 each) (Patent AU764632B2), although the absence of Neu5Gc-GM3 in the kidney from fetal autopsies was also reported (0/3) [34]. The weak staining of Neu5Gc-GM3 was observed in neurons from the fetal brain (1/3, 33.3%) but not in glial cells [16].

In normal adult tissues, Neu5Gc expression was primarily localized to epithelial cells and their secretions, as well as in blood vessels. Tangvoranuntakul et al. and Diaz et al. demonstrated this distribution using specific anti-Neu5Gc antibodies [30,31]. A Neu5Gc-containing mucin was detected in renal tubules, urinary bladder epithelium, sudoriferous glands, pneumocyte type II cells, thyroid gland epithelium, biliary ducts, pancreatic exocrine ducts, islets of Langerhans, and endocervix samples [35].

On the other hand, Neu5Gc-GM3 expression was limited to specific tissues and secretions. In a study conducted by Carr et al., which included 96 cases of frozen normal tissues and 14F7 mAb, the expression of Neu5Gc-GM3 was detected in breast secretion (3/4), as well as in the mucus cells from both small (5/5) and large intestines (7/7) [36]. Meanwhile, Blanco et al. only found this ganglioside in one-third (33.3%) of cases of the small intestine and its secretion, among other 94 normal tissues evaluated [16]. The expression of Neu5Gc-GM3 was also found in gastric glands from three-eighths (37.5%) of the stomach [37], as well as in the large intestine [23], using formalin-fixed and paraffin-embedded (FFPE) tissues. Additionally, Neu5Gc-GM3 was found in renal tubules (2/6) but not in renal corpuscles, corroborating results from other studies using anti-Neu5Gc antibodies [30,31,38].

Overall, Neu5Gc-containing glycoconjugates exhibit increased expression in fetal tissues, likely due to the heightened uptake associated with accelerated growth. In contrast, their occurrence in adult tissues is more restricted, predominantly in epithelial cells with high turnover rates [30]. Further research is necessary to elucidate the biological roles and implications of Neu5Gc-containing glycoconjugates in both fetal development and normal adult physiology.

### 2.2. Expression of Neu5Gc-Containing Glycoconjugates in Human Tumors

Neu5Gc-containing glycoconjugates have been consistently detected in a wide variety of human tumors (Table 1 and Table 2) but absent or limitedly expressed in normal tissues [6,14,17,39,40,41]. Using a polyclonal anti-Neu5Gc chicken antibody, Neu5Gc was identified in 5/8 (62.5%) breast carcinomas, 3/3 (100%) ovarian carcinomas, 2/4 (50%) prostate carcinomas, and 1/4 (25%) lung carcinomas [26,30], also confirmed by DMB derivatization and HPLC [26]. Additionally, Neu5Gc expression was evidenced in 6/12 (50%) colon and liver tumors and 4/8 (50%) spleen samples from lymphoma or leukemia patients using Hanganutziu–Deicher (HD) antibodies [39]. The HD antigen involves the Neu5Gc, forming part of glycoproteins or glycolipids [42,43].

The presence of Neu5Gc-containing mucins was observed in 37/37 (100%) breast carcinomas, 7/11 (63.6%) lung carcinomas, 3/4 (75%) renal carcinomas, 4/4 (100%) cervical carcinomas, and 4/4 (100%) endometrial carcinomas using the 3El-2 mAb [35], which was posteriorly confirmed in human endometrial tumors by electrospray mass spectrometry [44].

In a broader survey, membrane immunofluorescence staining with N-glycolylneuraminyllactosylceramide (HD3) chicken antiserum revealed Neu5Gc in 9/16 (56.3%) gastric adenocarcinomas, 8/14 (57.1%) breast adenocarcinomas, 3/12 (25%) colorectal tumors, 4/7 (57.1%) nasopharyngeal carcinomas, 2/3 (66.7%) uterine carcinomas, 5/10 (50%) leukemias, and 2/5 (40%) malignant lymphomas [45]. Neu5Gc-containing glycosphingolipids were also found in gastric and liver tumors, malignant lymphomas, and teratomas, as demonstrated by gas chromatography-mass spectrometry [40]. In addition, Hirabayashi et al. reported the expression of Neu5Gc-containing gangliosides in 6/11 (54.5%) of human melanoma tissues using thin layer chromatography (TLC) immunostaining [46].

Particularly, Neu5Gc-GM2 was strongly expressed in 14/16 (87.5%) colon carcinomas and 6/12 (50%) breast carcinomas [17]. Additional studies also found Neu5Gc-GM2 in 10/107 (9.3%) germ cell tumors [47]. Furthermore, Neu5Gc-GM3 and other gangliosides were detected in 7/16 (43.7%) colon cancers [14]. The aberrant expression of Neu5Gc-GM3 was also evidenced in 8/12 (66.7%) of breast carcinoma samples using mass spectrometry and TLC immunostaining [41]. Moreover, the presence of this ganglioside was detected in breast cancer (18/18) and cutaneous melanoma (10/10) by IHC using the 14F7 mAb [36].

On the other hand, Neu5Gc expression was demonstrated in breast, ovarian, and prostate carcinoma using FFPE tissues [31]. Similarly, Saida et al. reported Neu5Gc expression in 7/11 (63.6%) human melanomas samples by IHC using an anti-HD antibody and FFPE tissues [48]. Additionally, Neu5Gc-containing glycolipids were identified in 9/17 (52.9%) hepatocellular carcinomas using IHC and the highly sensitive tyramide signal amplification system, suggesting that the Neu5Gc antigen is not completely extracted from tissue samples during the histological processing [49]. In NSCLC, Neu5Gc-GD1a and Neu5Gc-GM3 were isolated from FFPE tissues and identified by TLC immunostaining with GMR8 mAb [6]. Moreover, studies have shown that the antigen recognized by 14F7 mAb is not entirely removed in these kind of samples [50,51].

The reactivity of 14F7 mAb was evidenced in breast cancer [52,53], cutaneous and mucosal melanoma [54,55], gastric, pancreatic, and liver cancers [37], colon adenocarcinoma [23,37], sarcomas [16,24], NSCLC [50,56], high-grade astrocytomas [16,57], cervical carcinoma [58], renal cell carcinoma and prostatic adenocarcinoma [38], urothelial carcinoma [53], and thyroid carcinoma [16] using FFPE tissues. Pediatric neuroectodermal tumors, such as Ewing sarcoma, neuroblastoma, and retinoblastoma, also expressed Neu5Gc-GM3 [34,59,60]. The presence of Neu5Gc-GM3 was also evidenced in many lymph nodes and another sites of metastasis [54,58]. Remarkably, in a recent report, the low reactivity of a similar 14F7 mAb was obtained in some of the aforementioned human tumors [61]. In this regard, a comparative study using both the original and the similar 14F7 mAb is suggested.

According to Wang et al., Neu5Gc accounts for 0.035% of the total sialic acid (0.03 µg/g) in throat cancer and lymph nodes, whereas Neu5Ac was the predominant sialic acid type (85 µg/g) in these tissue samples (about 97%) [62]. Moreover, in a mixed group of tumors, Neu5Gc ranged from 12.4 to 48.1 pmol/mg of glycoshingolipid in positive samples, which corresponded to 0.03–0.14% of total sialic acid, while Neu5Ac ranged from 9.7 to 57.8 nmol/mg of glycosphingolipid in the same series [40]. In particular, it was shown that between 1 and 17% of the total sialic acid in MUC1 mucin from breast cancer cells was N-glycolylated [63]. Using a sensitive TLC/immunostaining approach, it was reported that Neu5Gc-GM2 levels in colon cancer accounted for around 0.3–3% of the total lipid-bound sialic acid [32].

In summary, the accumulation of Neu5Gc in human tumors, contrasted with its limited presence in normal adult tissues, highlights its potential as a xeno-autoantigen. The role of Neu5Gc-containing glycoconjugates in tumor development and progression warrants further investigation, as discussed in subsequent sections.

**Table 1:** Expression of Neu5Gc-contanining glycoconjugates in fresh/frozen tumors (only studies with ≥ 10 cases evaluated).

Refs.	Molecule	Tissue Type	Methods	Neu5Gc Positivity Pos/Total (%)
[41]	Neu5Gc-GM3	Frozen tissues	MS/TLC	Breast carcinoma = 8/12 (66.7)
[36]	Neu5Gc-GM3	Frozen tissues	IHC with 14F7 mAb	Breast carcinoma = 18/18 (100) Cutaneous melanoma = 10/10 (100)
[14]	Neu5Gc-containing gangliosides	Frozen tissues	TLC	Colon adenocarcinoma = 7/16 (43.7)
[17]	Neu5Gc-GM2	Frozen tissues	IHC	Colon adenocarcinoma = 14/16 (87.5) Breast carcinoma = 6/12 (50.0)
[47]	Neu5Gc-GM2	Frozen tissues	IHC with MK2-34 mAb	Germ cell tumors = 10/107 (9.3)
[49]	Neu5Gc	Frozen tissues	IHC with Hu/Ch 2-7 and 6-1 mAbs	Hepatocellular carcinoma = 9/17 (52.9)
[46]	Neu5Gc-containing gangliosides	Frozen tissues	TLC	Cutaneous melanoma = 6/11 (54.5)
[45]	HD3	Cells from fresh tissues	MIT/TLC	Gastric adenocarcinoma = 9/16 (56.3) Breast carcinoma = 8/14 (57.1) Colorectal tumor = 3/12 (25.0) Leukemia = 5/10 (50.0)
[51]	Neu5Gc-GM3	Frozen tissues	IHC with 14F7 mAb	Lung carcinoma = 18/18 (100)

Legend: MS, mass spectrometry; TLC, thin layer chromatography; IHC, immunohistochemistry; MIT, membrane immunofluorescence test; and HD3, N-glycolylneuraminyllactosyl-ceramide.

**Table 2:** Expression of Neu5Gc-contanining glycoconjugates in FFPE tumors.

Refs	Molecule	Methods	Neu5Gc Positivity. Pos/Total (%)
[6]	Neu5Gc-containing gangliosides	IHC with GMR8 mAb	NSCLC = 86/93 (93.5)
[33]	Neu5Gc-containing gangliosides	IHC with P3 mAb	Breast carcinoma = 12/12 (100)
[48]	Neu5Gc	IHC with anti-HD Ab	Cutaneous melanoma = 7/11 (63.6)
[37]	Neu5Gc-GM3	IHC with 14F7 mAb	Esophageal tumors = 5/15 (33.3) Gastric adenocarcinoma = 12/12 (100) Colon adenocarcinoma = 12/12 (100) Pancreatic adenocarcinoma = 11/11 (100) Hepatocellular carcinoma = 13/14 (92.9)
[54]	Neu5Gc-GM3	IHC with 14F7 mAb	Basocellular carcinoma = 2/13 (15.4) Cutaneous melanoma = 28/28 (100)
[35]	O-linked mucin-containing Neu5Gc	IHC with 3E1-2 mAb	Breast carcinoma = 37/37 (100) Lung carcinoma = 7/11 (63.6)
[38]	Neu5Gc-GM3	IHC with 14F7 mAb	Renal cell carcinoma = 11/36 (30.5) Prostatic adenocarcinoma = 17/20 (85)
[64]	Neu5Gc-GM3	IHC with 14F7 mAb	Lymphoma = 33/37 (89.2) Cutaneous melanoma (LNM) = 15/17 (88.2) Breast carcinoma (LNM) = 14/14 (100) Colon adenocarcinoma (LNM) = 9/12 (75.0)
[65]	Neu5Gc-GM3	IHC with 14F7 mAb	Nasopharyngeal carcinoma = 13/14 (92.8)
[50]	Neu5Gc-GM3	IHC with 14F7 mAb	NSCLC = 84/90 (93.3)
[56]	Neu5Gc-GM3	IHC with 14F7 mAb	NSCLC = 155/165 (93.9)
[51]	Neu5Gc-containing ganglioside	IHC with P3 mAb	Lung carcinoma = 35/36 (97.2)
[23]	Neu5Gc-GM3	IHC with 14F7 mAb	Colon adenocarcinoma = 50/50 (100)
[24]	Neu5Gc-GM3	IHC with 14F7 mAb	Sarcoma = 50/50 (100)
[16]	Neu5Gc-GM3	IHC with 14F7 mAb	Astrocytoma * = 10/19 (52.6) Sarcoma = 25/30 (83.3) Thyroid carcinoma = 23/25 (92.0)
[52]	Neu5Gc-GM3	IHC with 14F7 mAb	Breast carcinoma = 126/126 (100)
[34]	Neu5Gc-GM3	IHC with 14F7 mAb	Wilms tumor * = 22/25 (88.0)
[59]	Neu5Gc-GM3	IHC with 14F7 mAb	Neuroectodermal tumor * = 23/27 (85.2)
[60]	Neu5Gc-GM3	IHC with 14F7 mAb	Retinoblastoma * = 21/21 (100)
[55]	Neu5Gc-GM3	IHC with 14F7 mAb	Oral mucosal melanoma = 37/44 (84.1)
[58]	Neu5Gc-GM3	IHC with 14F7 mAb	Cervical carcinoma = 28/29 (96.5) Astrocytoma = 24/45 (53.3)

Legend: LNM, lymph node metastasis; IHC, immunohistochemistry; NSCLC, non-small cell lung carcinoma; and * Pediatric samples.

## 3. Potential Mechanisms Implicated in Neu5Gc Expression in Human Tissues

The expression of Neu5Gc in human tissues is an anomaly, given that humans lack the enzymatic ability to synthesize this molecule due to a genetic deletion. The enzyme cytidine monophosphate-N-acetylneuraminic acid hydroxylase (CMAH), encoded by the *CMAH* gene, converts Neu5Ac to Neu5Gc in other species. However, in humans, a 92 bp deletion in the *CMAH* gene results in a truncated, non-functional protein, making Neu5Ac the predominant sialic acid in human glycoconjugates [6,13,66,67]. Despite this, Neu5Gc is consistently detected in malignant tumors [35,36,45], with only a limited presence in normal tissues [30,36]. This discrepancy suggests alternative mechanisms for Neu5Gc expression in human cells.

One major pathway for the presence of Neu5Gc in human tissues involves its uptake and incorporation from external sources, primarily dietary intake. Neu5Gc is abundant in red meat (e.g., lamb, pork, and beef) and dairy products (e.g., goat and sheep milk products), and its integration into human glycoproteins has been well documented. Increased amounts of mucins containing Neu5Gc were detected in humans after the oral administration of purified Neu5Gc from porcine submaxillary mucin. Moreover, it was demonstrated that human cells can absorb free Neu5Gc from these dietary sources and incorporate it into glycoproteins, as demonstrated by Western blot analyses using anti-Neu5Gc antibodies [30]. Neu5Gc enters cells via clathrin-independent endocytosis, primarily through bulk-phase pinocytosis [68]. Once internalized, Neu5Gc is processed by lysosomal enzymes, such as neuraminidase-1 and the lysosomal sialic acid transporter sialin, enabling its integration into glycoproteins using the same enzymatic machinery that processes Neu5Ac [68,69]. Interestingly, malignant cells show an enhanced ability to accumulate Neu5Gc compared to normal cells. In fact, under nutrient-deprived conditions, cancer cells treated with exogenous Neu5Gc exhibit a three- to six-fold increase in membrane-bound Neu5Gc compared to normal cells, which show only minimal uptake [70]. This selective incorporation suggests a link between the altered metabolism of tumor cells and their ability to utilize extracellular Neu5Gc.

Hypoxia, a common feature of the tumor microenvironment, further amplifies Neu5Gc uptake and expression. In fact, Yin et al. demonstrated that hypoxia induces the expression of sialin in human malignant cells and enhances the uptake of Neu5Gc from the external medium and its posterior incorporation to newly synthesized Neu5Gc-GM2 ganglioside. Additionally, the authors found colocalization of sialin and Neu5Gc-GM2 in hypoxic regions away from tumor vasculature [17]. Also, studies conducted by Bousquet et al. and Dorvignit et al. revealed that hypoxia induces Neu5Gc-GM3 expression in HeLa cells and SKOV3 ovarian cancer cells, as detected by flow cytometry with the 14F7 mAb [18,19]. Notably, tumor hypoxia also induces the transcription of genes involved in glycan synthesis, such as the sialyltransferase *ST3Gal-I*, which is involved in the sialylation of glycolipids and glycoproteins [12,71]. Taken together, these hypoxia-driven mechanisms may favor the expression of Neu5Gc-containing glycoconjugates, particularly in cancer cells, through the enhanced cellular uptake of Neu5Gc from the extracellular environment and glycan synthesis. Elevated Neu5Gc levels in the sera of cancer patients provide an abundant external source for tumor cells to exploit [72,73,74].

Although humans lack the CMAH enzyme, some studies propose alternative mechanisms for Neu5Gc production. Bai et al. suggested that reactive oxygen species (ROS) may mediate the conversion of Neu5Ac to Neu5Gc under oxidative stress, particularly in lung cancer cells [75]. Similarly, Bousquet et al. identified subunit B of the succinate dehydrogenase complex, an iron–sulfur [Fe2S2]-containing protein, as a possible source for Neu5Gc synthesis during hypoxia [18].

While Asakawa et al. observed the Neu5Gc expression in K562 leukemia cells cultured in serum-free media [76], definitive evidence for de novo Neu5Gc synthesis in human cells remains lacking [19]. These hypotheses remain unproven, with dietary incorporation and tumor-specific metabolic alterations as the primary explanations for the Neu5Gc presence in human tissues.

## 4. Contribution of Neu5Gc to Tumor Progression

Neu5Gc differs from Neu5Ac by a single oxygen atom at the C-5 position [4]. This minor structural change significantly alters the biological properties of sialic acid-containing glycoconjugates. Neu5Gc-containing gangliosides were absent in some human melanoma cells cultured in a serum-free medium. However, when these cells are grown as xenografts in athymic (nu/nu) mice, they express increased levels of Neu5Gc-containing gangliosides, including Neu5Gc-GM3, Neu5Gc-GM2, NeuAc-NeuGc-GD3, NeuGc-NeuAc-GD3, and NeuGc-NeuGc-GD3 [77]. Similarly, an increase in Neu5Gc-containing gangliosides was observed in U87-MG human glioma xenografts in severe combined immunodeficiency (SCID) mice compared with in vitro cultured cells [78]. Moreover, Labrada et al. reported a shift from Neu5Ac to Neu5Gc in GM3 ganglioside composition from primary tumors to metastatic lesions in the 3LL-D122 Lewis lung carcinoma spontaneous metastasis murine model [79]. These findings suggest the potential involvement of Neu5Gc-containing glycoconjugates in cancer progression.

Treatment with Neu5Gc has been shown to promote the proliferation of both colorectal cancer cells and normal intestinal epithelial cells, associated with increased levels of HRAS, CCNA2 (Cyclin A2), and AKT2 proteins [80]. Similarly, a study in A431 epithelial cells revealed that Neu5Gc-GM3 was less effective at inhibiting epidermal growth factor receptor (EGFR) phosphorylation compared to Neu5Ac-GM3 [6]. The preincubation of B16 (melanoma) and F3II (mammary carcinoma) cells with Neu5Gc reduced the time to tumor appearance and increased both the number and size of lung metastases in C57BL/6 and Balb/c mice, respectively [21]. Moreover, the exogenous incorporation of Neu5Gc into Lewis lung carcinoma 3LL-D122 cells was associated with the increased formation of lung tumor nodules and a higher number of macronodules (>2 mm in diameter) [81]. In Apc Min/+ mice, Neu5Gc treatment increased mRNA levels of pro-inflammatory and immunomodulatory genes such as *TNF*, *CD80*, *IL-17F*, and *CCL17* [82].

Conversely, decreasing Neu5Gc-containing glycoconjugates can inhibit tumor growth. Inhibiting ganglioside synthesis in P3 × 63 cells using a glucosylceramide synthase inhibitor (D-PDMP) reduced tumor growth in Balb/c mice inoculated subcutaneously ([22,83]. Notably, in these cells, Neu5Gc-GM3 is the major ganglioside, with a Neu5Gc-GM3 to Neu5Ac-GM3 ratio of 85:15 [84]. Similarly, knocking down the CMAH enzyme in L1210 cells significantly diminished both their capacity for anchorage-independent growth and their ability to develop subcutaneous tumors compared with L1210 wild-type cells [85]. Furthermore, the transfection of B16 cells with CMAH increased proliferation rates and cell adhesion properties compared to parental cells; however, these *CMAH*-transfected cells exhibited decreased tumorigenicity in comparison with parental cells [86].

One possible explanation for the opposite results regarding the role of Neu5Gc expression in tumor growth is related to the molecular mechanisms by which malignant cells metabolize and express Neu5Gc on the cell surface, which may alter its biological function [21,86]. The uptake and reuse of Neu5Gc through sialin may economize the cell metabolic energy required for de novo sialic acid synthesis [87]. Meanwhile, the activity of CMAH forces tumor cells to convert CMP-Neu5Ac into CMP-Neu5Gc, disrupting the cell biology and potentially impairing the tumor formation [86]. Other potential causes could be related to an effective host immune response against tumor cells expressing elevated levels of Neu5Gc-containing molecules on the plasmatic membrane. The role of Neu5Gc expression and anti-Neu5Gc antibody response in tumor growth will be discussed in the next paragraphs. The capacity of anti-Neu5Gc antibodies to enhance or inhibit the growth of Neu5Gc-positive tumors has also been reported. In Neu5Gc-deficient mice (*CMAH*−/−), the combination of dietary Neu5Gc intake and treatment with anti-Neu5Gc antibodies increased the incidence of hepatocellular carcinoma. This effect was related to elevated levels of IL-6 in peritoneal fluids, serum acute-phase proteins (APPs), and haptoglobin, linking anti-Neu5Gc-dependent inflammation with cancer development [25]. The treatment of Neu5Gc-deficient mice bearing Neu5Gc-expressing tumors induced by B16 cells with anti-Neu5Gc antibodies increased the infiltration of inflammatory cells and vascular density. This was associated with elevated levels of cyclooxygenase-2 (COX-2), which is linked to multiple pro-inflammatory pathways, and the upregulation of the vascular endothelial growth factor (VEGF) [88,89]. The ability of human anti-Neu5Gc antibodies to enhance tumor growth was also demonstrated in *CMAH*−/− mice using the Neu5Gc-expressing MC38 colon carcinoma model [26].

Humans have variable amounts of circulating IgA, IgM, and IgG antibodies against Neu5Gc [30,90]. These anti-Neu5Gc antibodies can recognize Neu5Gc present in human tissues [91]. The specificity, levels, and repertoires of the anti-Neu5Gc IgG immune response can be influenced by factors such as a Neu5Gc-enriched diet, gender, and age [92]. Interestingly, the use of intermediate dosages of affinity-purified anti-Neu5Gc human antibodies increased the weight of MC38 tumors in *CMAH*−/− mice, while high levels of these antibodies tended to inhibit tumor growth [26]. These observations suggest that high levels of anti-Neu5Gc antibodies could induce an effective anti-tumor response, whereas inadequate immune responses against Neu5Gc could lead to chronic inflammation and potentially to cancer development.

**Figure 2:**
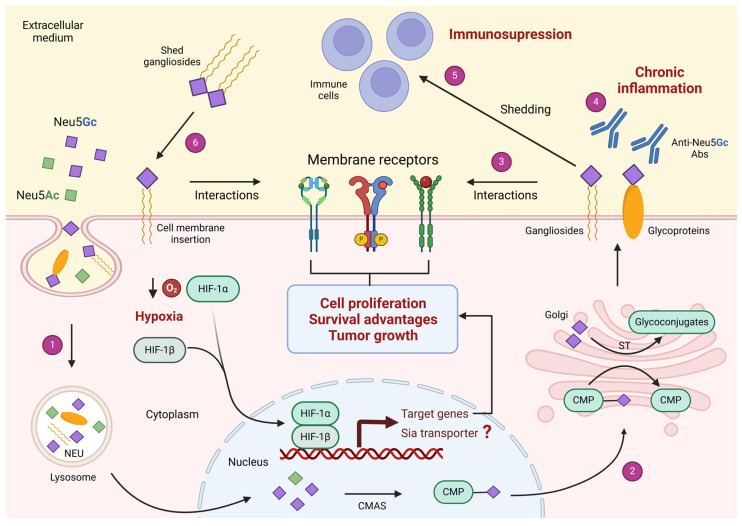
A proposed model of Neu5Gc glycoconjugate contribution to cancer progression: 1: Neu5Gc is uptaken for human malignant cells from the extracellular medium, which may be potentiated by hypoxia. 2: Neu5G is incorporated into newly synthesized glycoconjugates and expressed on the surface of tumor cells. 3: On the cell surface, Neu5Gc glycoconjugates could interact with membrane receptors, promoting cell proliferation and survival. 4: A consistent inadequate humoral immune response against Neu5Gc could promote chronic inflammation. 5: Neu5Gc-containing gangliosides could also be shedding to the extracellular medium, inducing local immunosuppression. 6: The shed Neu5Gc-containing gangliosides can be inserted in the membrane of adjacent cells and then interact with cell surface receptors. These facts together could contribute to tumor growth and progression. NEU, neuroaminidase; CMAS, cytidine monophosphate N-acetylneuraminic acid synthetase; ST, sialyltransferase. Created by BioRender.com (accessed on 6 January 2025).

In summary, clinical and experimental evidence indicates that Neu5Gc may contribute to the initiation and progression of human tumors by activating molecular pathways involved in cancer biology and facilitating immune evasion. A hypothetical model illustrating the potential role of Neu5Gc-containing glycoconjugates is shown in Figure 2.

## 5. Role of Neu5Gc-GM3 in Tumor Biology: A Case Study

Neu5Gc-GM3 ganglioside is among the most extensively studied Neu5Gc-containing glycoconjugates concerning its role in human tumor development and progression. This focus stems largely from the antitumor responses observed in cancer patients treated with immunotherapeutic agents targeting this molecule [27,29,36,93,94,95].

### 5.1. Neu5Gc-GM3 Expression and Tumor Aggressiveness

The aberrant expression of Neu5Gc-GM3 has been linked to advanced stages of colon cancer. Its presence correlated with decreased overall survival in patients, as indicated by both univariate (*p* = 0.0078; log-rank test) and multivariate analyses (hazard ratio [HR] = 0.268; 95% confidence interval [CI] 0.078–0.920; and *p* = 0.036) [23]. In sarcomas, a high Neu5Gc-GM3 expression was associated with older patient age (*p* = 0.014), advanced clinical stages (*p* = 0.022), higher malignancy grades (*p* = 0.013), and increased proliferation rates (*p* = 0.012). Consequently, Neu5Gc-GM3 expression serves as a significant prognostic factor for overall survival (*p* = 0.034) [24].

Similarly, a significant increase in Neu5Gc-GM3 occurrence has been observed in high-grade astrocytomas compared to low-grade astrocytic tumors (78.6% vs. 10.0%; *p* = 0.026). The differential accumulation of this ganglioside has also been noted in transitional cell carcinoma of the urinary bladder, with expression rates of 94.9%, 77.8%, and 63.8% for grade III, II, and I tumors, respectively (*p* = 0.042) [53]. Later, aberrant Neu5Gc-GM3 expression was associated with an increased nuclear grade in breast carcinoma [52], a parameter traditionally linked to higher proliferation rates and breast cancer progression [96,97].

In NSCLC, high Neu5Gc-GM3 expression correlated with increased proliferation rates. Elevated levels of this ganglioside were also associated with poorer 5-year overall survival (OS) (*p* = 0.020; log-rank test), and its presence was an independent prognostic factor in multivariate analysis (HR = 3.394; 95% CI 1.342–8.584; and *p* = 0.010) [50]. Additionally, the increased expression of Neu5Gc-containing gangliosides (Neu5Gc-GM3 and Neu5Gc-GD1a) was related to shorter progression-free survival in NSCLC patients (47.5 vs. 56.4 months; *p* < 0.01) [6]. Opposite results were reported by Van Cruijsen et al. [56].

On the other hand, patients with NSCLC exhibiting both Neu5Gc-GM3 and EGFR positivity show significantly decreased OS compared to those with Neu5Gc-GM3-positive but EGFR-negative tumors (40.0% vs. 68.2%; *p* < 0.000) [50]. Furthermore, among Neu5Gc-GM3+/EGFR+ tumors, an additional expression of the EGF ligand was associated with the poorest 5-year OS rates compared to those lacking either the EGF or EGFR expression (25.0% vs. 66.7% vs. 80.0%; *p* < 0.000) [50]. In malignant astrocytomas, the co-expression of Neu5Gc-GM3 and the EGFR was significantly higher in high-grade tumors (III–IV), which are characterized by increased proliferation and aggressiveness. No such co-expression was observed in low-grade astrocytoma (I–II) [58].

### 5.2. Neu5Gc-GM3 Expression and Immune Response

The shedding of immunosuppressive gangliosides from malignant cell membranes into the tumor microenvironment has been documented [98]. It was also reported that tumor cell gangliosides can be shed in three forms: membrane vesicles, micelles, and monomers [99,100]. Remarkably, it was found that > 90% of shed gangliosides were in monomeric form and only < 10% in micelles [99]. These circulating gangliosides can integrate into adjacent cells by inserting their lipid anchors into the plasma membrane [101]. Scursoni et al. suggested that tumor cells may shed Neu5Gc-GM3 to nearby non-tumor tissues [34,59]. Additionally, the incorporation of shed Neu5Gc-GM3 into lymphocytes surrounding metastatic tumor masses in lymph nodes has been proposed [37]. This mechanism may enable malignant cells to evade effective immune responses, facilitating cancer progression.

De León et al. demonstrated that Neu5Gc-GM3 can reduce CD4 expression in both mouse and human T lymphocytes by inserting into their plasma membranes [22]. Exposure to Neu5Gc-GM3 decreased CD4 levels in CD4 + CD25− T cells and naturally occurring CD4 + CD25+ regulatory T cells [83]. Moreover, Neu5Gc-GM3 reduced the proliferation of CD4 + CD25− T cells and shifted cytokine production, decreasing interferon gamma while increasing IL-4 and IL-10 [83]. In NSCLC samples, higher Neu5Gc-GM3 levels were associated with a decreased proportion of mature CD83+ dendritic cells (DCs) [56]. Notably, Neu5Gc-GM3 impaired the differentiation and maturation of bone marrow-derived DCs induced by lipopolysaccharides [83]. Reduced DC levels disrupt effective tumor-specific immune responses, promoting cancer progression and metastasis [102,103,104].

Conversely, as Neu5Gc is immunogenic in humans, antibodies against Neu5Gc-containing glycoconjugates may play a crucial role in tumor immune surveillance [105]. Healthy individuals possess anti-Neu5Gc-GM3 antibodies capable of binding to and killing tumor cells expressing this molecule [105]. It has been shown that CD1d binds to Neu5Gc-GM3 and that human B lymphocytes present this ganglioside to invariant natural killer T (iNKT) cells in a CD1d-dependent manner [106]. iNKT cells facilitate B-lymphocyte proliferation and antibody production [107], potentially contributing to the pool of anti-Neu5Gc-GM3 antibodies in human sera.

Rodríguez-Zhurbenko et al. reported an increased proportion of circulating human B1 lymphocytes in healthy individuals, which produce IgM antibodies against Neu5Gc-GM3 [108]. B1 cells are a subset of B cells capable of secreting natural antibodies [109]. However, both the percentage of B1 cells and their capacity to secrete IgM decrease with age [110]. Correspondingly, a decline in anti-Neu5Gc-GM3 responses has been observed with increasing age in healthy donors [108]. Notably, significantly lower levels of anti-Neu5Gc-GM3 IgM were found in the sera of lung cancer patients compared to age- and gender-matched healthy individuals [105]. This deficit may impair effective immune responses against Neu5Gc-GM3-expressing tumor cells, favoring tumor progression.

### 5.3. Antitumor Activity of Therapeutic Strategies Targeting Neu5Gc-GM3

Therapies targeting Neu5Gc-GM3 have demonstrated clinical benefits across various malignancies. In a phase I/II clinical trial involving patients with metastatic cutaneous melanoma, vaccination with NGcGM3/VSSP induced specific anti-Neu5Gc-GM3 IgM and IgG antibodies in all treated patients (n = 34), achieving disease control in 38.46% of cases [28]. The NGcGM3/VSSP vaccine elicits antibodies capable of binding to and killing Neu5Gc-GM3-overexpressing cells [111]. Treatment with NGcGM3/VSSP also showed survival benefits in metastatic breast cancer patients, particularly those with non-visceral metastases compared to the control group (26.17 vs. 12.17 months; *p* = 0.0493). The lack of reactivity of post-immune sera against Neu5Ac-GM3 or Neu5Ac-GM2 supported the specificity of the humoral response against the immunizing antigen [93]. Although naturally occurring anti-Neu5Gc-GM3 antibodies were found in some pre-immune sera from breast cancer patients, the titer of these antibodies was significantly lower compared to those induced with vaccination [94]. In a phase III clinical trial involving stage II–III breast cancer patients, the cytotoxic activity of hyperimmune sera correlated with better disease-free survival in patients with four or more lymph node metastases (HR = 0.26; 95% CI: 0.06–1.06; and *p* = 0.044) [94]. Hyperimmune sera also prevented the reduction in CD4 expression on T lymphocytes induced by Neu5Gc-GM3 [93,94].

Promising results have been obtained with Vaxira^®^ (racotumomab-alum vaccine), an anti-idiotypic vaccine that induces an immune response against Neu5Gc-GM3 [112]. In a phase I clinical trial, survival advantages were observed in small cell lung carcinoma (SCLC) patients treated with Vaxira^®^, along with the demonstrated reactivity of post-immune sera against SCLC tissue sections [113]. In a clinical trial using racotumomab-alum as switch maintenance therapy in stage IIIB/IV NSCLC patients, the treated group showed benefits in both OS (8.23 vs. 6.80 months; *p* = 0.004) and progression-free survival (5.33 vs. 3.90 months; *p* = 0.039) compared to the placebo group. The ability of hyperimmune sera to bind to and kill ≥30% of Neu5Gc-expressing L1210 cells was associated with increased OS [29]. Notably, hyperimmune sera from immunized NSCLC patients were shown to recognize and kill tumor cells expressing this ganglioside through complement-independent mechanisms [29,112].

In NSCLC patients immunized with racotumomab-alum, the capacity of post-immune sera to specifically react to Neu5Gc-GM3 was confirmed by ELISA and HPTLC, while no reactivity against the closely related Neu5Ac-GM3 was detected [112]. In the same way, post-immune sera from melanoma patients immunized with racotumumab were not able to bind to B16 murine melanoma cells, which express high levels of Neu5Ac-GM3 but no Neu5Gc-GM3 [114]. Moreover, the capacity of generated antibodies to recognize the Neu5Gc-GM3-positive L1210 cells was demonstrated. But these antibodies were not able to react against L1210 CMAH-kd cells, which lack Neu5Gc-sialoconjugates. Furthermore, the cytotoxicity of hyperimmune sera was significantly reduced against L1210 cells treated with d-threo-PDMP, an inhibitor of glucosylceramide synthetase [29]. Taken together, these facts suggest that post-immune sera bind to Neu5Gc-GM3, although the authors cannot deny the potential recognition of other Neu5Gc-containing glycosphingolipids.

Recently, the efficacy of 14F7 chimeric antigen receptor (CAR) T cells in binding to and eliminating human tumor cells expressing Neu5Gc-GM3 was demonstrated following the introduction of the murine *CMAH* gene [115]. Encouraging results have also been reported in NSCLC patients treated with Vaxira^®^ in combination with CIMAvax-EGF [116]. CIMAvax-EGF is a molecular cancer vaccine that induces an immune response against circulating the epidermal growth factor (EGF) ligand [117]. The survival rate in advanced NSCLC patients unresponsive to first-line chemotherapy was 40% at one year after treatment with these vaccines. This clinical benefit is comparable to that reported with second-line chemotherapy but with significantly reduced toxicity [116]. Overall, the survival advantages provided by molecular cancer vaccines targeting Neu5Gc-GM3 support the continued use of this molecule as a target for cancer immunotherapy.

## 6. Conclusions

The role of Neu5Gc in human tumor development and progression remains a topic of significant interest. In this review, we explored the molecular mechanisms by which Neu5Gc may enter human cells and examined both epidemiological and experimental evidence linking Neu5Gc expression to human cancer.

Neu5Gc-containing glycoconjugates are minimally expressed in fetal and normal adult tissues—limited to cells with high turnover—but they preferentially accumulate in human tumors and their metastases. Although some molecular pathways for the de novo synthesis of Neu5Gc in human cells have been proposed, the most widely accepted hypothesis is that Neu5Gc is acquired from the diet, a process enhanced by the hypoxic conditions prevalent in tumors.

The current literature suggests that Neu5Gc plays a multifaceted role in human carcinogenesis. Firstly, Neu5Gc-containing glycoconjugates may interact with cell surface receptors, promoting cell proliferation and survival. Secondly, Neu5Gc-containing gangliosides can be shed from tumor cells into the extracellular environment, inducing immunosuppression. Thirdly, an inadequate immune response involving specific antibodies generated against Neu5Gc may lead to chronic inflammation, further contributing to cancer progression.

Further studies are needed to elucidate the precise role of Neu5Gc-containing glycoconjugates in tumor biology. A deeper understanding of these mechanisms could potentially lead to the development of novel therapeutic strategies targeting Neu5Gc-related pathways.

## Data Availability

Not applicable.

## Supplementary Materials

The following supporting information can be downloaded at https://www.mdpi.com/article/10.3390/biom15020253/s1, Supplementary Figure S1: Scheme of ganglioside structure and synthetic pathway.
