# Supplementary material for: The Role of Non-Human Sialic Acid Neu5Gc-Containing Glycoconjugates in Human Tumors: A Review of Clinical and Experimental Evidence
Source: Biomolecules. 2025 Feb 10;15(2):253. doi: 10.3390/biom15020253 (PMC11853303; doi:10.3390/biom15020253)
Supplement: Supplementary file 1 [file biomolecules-15-00253-s001.zip › biomolecules-3377971-supplementary.pdf]

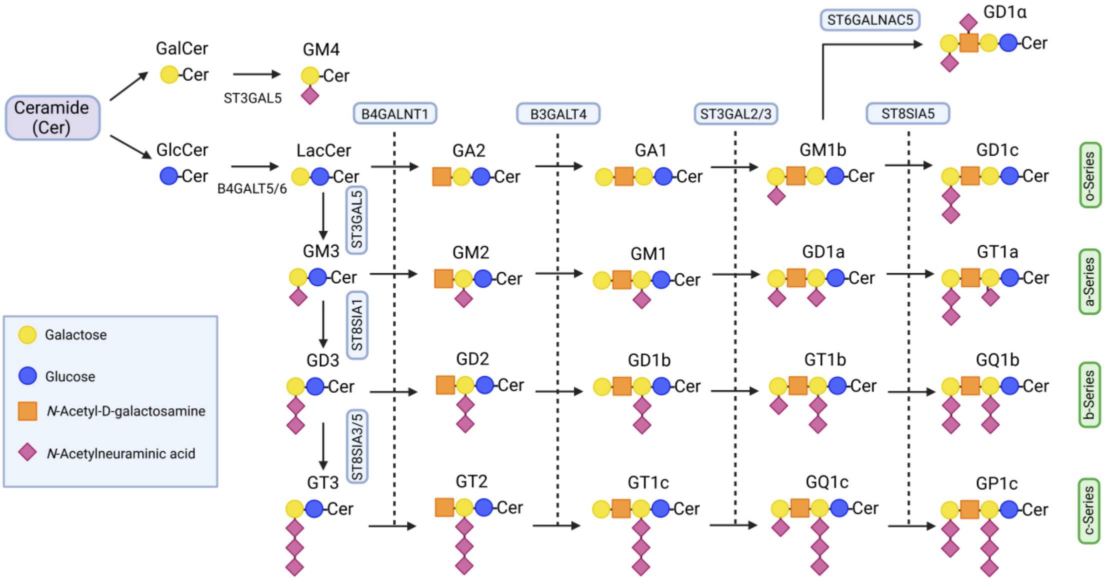

Supplementary Figure S1. Scheme of gangliosides structure and synthetic pathway. These molecules are synthesised from LacCer or GalCer precursors throught the addition of sialic acids catalyzed by different sialyltransferases (STs). Cer, ceramide; Gal, galactose; Glc, glucose; Lac, lactosyl; ST3GAL5, ST3 βgalactoside α-2,3-sialyltransferase 5; B4GAL5/6, β-1,4-Galactosyltransferase 5 and 6; ST8SIA1, ST8 α-N-acetyl-neuraminide α-2,8-sialyltransferase 1; ST8SIA3/5, ST8 α-N-acetyl-neuraminide α-2,8-sialyltransferase 3 and 5; B4GALNT1, β-1,4-N-acetylgalactosaminyltransferase 1; B3GALT4, β-1,3-galactosyltransferase 4; ST3GAL2/3, ST3 βgalactoside α-2,3-sialyltransferase 2 and 3; ST6GALNAC5, α-N-acetylgalactosaminide α-2,6-sialyltransferase 5.
